# *Lacticaseibacillus rhamnosus* LRa05 mediates dynamic regulation of intestinal microbiota in mice with low-dose DSS-induced chronic mild inflammation

**DOI:** 10.3389/fmicb.2024.1483104

**Published:** 2024-10-08

**Authors:** Yao Dong, Zhonghui Gai, Mei Han, Yunjiao Zhao

**Affiliations:** ^1^Department of Research and Development, Wecare Probiotics Co., Ltd., Suzhou, China; ^2^Department of Food Quality and Safety, Shanghai Business School, Shanghai, China; ^3^College of Life Science and Technology, Huazhong Agricultural University, Wuhan, China

**Keywords:** chronic inflammation, dextran sulfate sodium, gut microbiota, probiotics, *Lacticaseibacillus rhamnosus*

## Abstract

**Aim:**

This study aimed to investigate the effects of low-dose dextran sulfate sodium (DSS) on the induction of chronic mild inflammation in mice and to evaluate the therapeutic potential of *Lacticaseibacillus rhamnosus* LRa05 (LRa05) to ameliorate the associated effects. The focus was on investigating changes in inflammatory, gut microbiota, serum lipopolysaccharide (LPS) and inflammatory cytokines.

**Methods:**

Mice were exposed to a low-dose of DSS to induce chronic mild inflammation and LRa05 was administered as a probiotic intervention. The experiment included determination of body weight, colon length, histological examinations, and analysis of LPS and inflammatory cytokines in serum over 12 weeks. In addition, liver function, oxidative stress and intestinal microbiota were examined to understand the comprehensive effects of DSS and LRa05.

**Results:**

Low-dose DSS did not lead to significant changes in body weight, colon length or histologic signs of inflammation. However, it led to a significant increase in serum levels of LPS, tumor necrosis factor-alpha (TNFα) and interleukin-6 (IL6). Intervention with LRa05 effectively attenuated these changes, particularly by lowering LPS levels and normalizing inflammatory cytokines. In addition, LRa05 protected against DSS-induced liver function damage and attenuated oxidative stress in the liver. Analysis of the gut microbiota demonstrated dynamic regulatory effects, where LRa05 intervention led to significant shifts in microbial populations, promoting a balanced microbiota profile. These changes are indicative of dynamic regulation by LRa05 in response to chronic mild inflammation, highlighting the probiotic’s role in modulating the gut environment.

**Conclusion:**

The LRa05 intervention showed multi-layered regulation in the chronic mild inflammation model by reducing inflammatory cytokines, maintaining liver function and restoring the balance of the gut microbiota. This provides experimental support for the potential use of LRa05 in chronic inflammation-related diseases and emphasizes the importance of probiotics for overall health. The study suggests that LRa05 is a potential therapeutic agent for the treatment of chronic inflammation associated with gut dysbiosis.

## 1 Introduction

In this dynamic field of gastrointestinal research, the intricate interplay between the gut microbiota and the health of the host has become a focal point, providing deep insights into the etiology of inflammation and metabolic disorders. The intestinal microbiota is closely linked to the development of diseases that affect immune regulation, metabolic regulation and the barrier function of the intestinal mucosa (Huang et al., 2023). Dysbacteriosis can lead to abnormal activation of the immune system and is associated with inflammatory diseases such as inflammatory bowel disease. Particularly, this study focuses on the impact of chronic mild inflammation induced by a low-dose of dextran sulfate sodium (DSS) and its modulation by the gut microbiota.

DSS is a compound with very low bioavailability that has a direct toxic effect on intestinal epithelial cells, leading to intestinal barrier disruption (Kiesler et al., 2015; Jackson et al., 2022), with high doses (2–3.5%) inducing acute colitis, while low concentrations (0.5–1%) induce chronic colitis (Osburn et al., 2007; Dong et al., 2022). This damage elicits typical ulcerative colitis (UC) symptoms and provides a powerful tool for the study of inflammatory bowel disease. Although the DSS model at high concentrations allows the simulation of severe colitis, fewer studies have been performed with low DSS concentrations. Picard et al. investigated low-grade inflammation induced by 0.5% DSS – and its therapeutic targets (Picard et al., 2019). Treatment with 0.25% DSS increased the colonization of invasive *Escherichia coli* adherent to Crohn’s disease in the intestine of mice and induced inflammation (Carvalho et al., 2009). Administration of 0.2% DSS to ApoE ^−/−^ mice did not result in signs and symptoms of colitis, but increased intestinal permeability to dextran fluorescein isothiocyanate and increased levels of markers of liver damage and insulin resistance in the blood (Mencarelli et al., 2012). 0.5% DSS induced a highly sensitive colitis model in mice fed a fiber-free diet (Shen et al., 2021). This model provides a unique platform to study the temporal dynamics of inflammation and its impact on the complex ecosystem of the gut microbiota.

Probiotics, as beneficial microorganisms, play a positive role in improving gut health by maintaining the balance of the microbiota, regulating immune responses and promoting barrier function (Papadopoulou et al., 2023). Probiotics exert a regulatory influence on the immune system, which is closely associated with their capacity to modulate the intestinal microbiota and metabolites. These beneficial microorganisms can stimulate the synthesis of short chain fatty acids, therefore supplying energy to intestinal epithelial cells and helping to regulate intestinal inflammation (Lukasova et al., 2011). Intervention with *Lactobacillus acidophilus* LA85 improved the diversity of gut microbiota, repaired damaged intestinal mucosa, and enhanced intestinal immunity in immunocompromised mice (Xue et al., 2022). *Bifidobacterium animalis* subsp. *lactis* BLa80 reduced intestinal inflammation and improved gut microbiota in a mouse model of ulcerative colitis induced by DSS (Dong et al., 2022).

Previous studies have shown that intervention with *Lacticaseibacillus rhamnosus* LRa05 significantly reduces insulin resistance, improves insulin sensitivity and modulates hepatic oxidative stress in mice with type 2 diabetes. In addition, the application of LRa05 also attenuates the inflammatory response induced by metabolic lipopolysaccharides and thus supports the maintenance of microecological balance in the gut (Sun et al., 2020; Wu et al., 2021). Research conducted on healthy individuals has also revealed that LRa05 can increase the abundance of beneficial gut bacteria and reduce the abundance of opportunistic pathogens (Gai et al., 2023). The aim of this study was twofold: First, to reveal the temporal dynamics of chronic mild inflammation induced by a low-dose of DSS and gain insight into the subtle changes in the composition of the gut microbiota; and second, to evaluate the therapeutic potential of LRa05 in modulating these changes. It is expected that this line of research will provide more detailed information for the field of gastrointestinal research and promote the development of probiotics in translational applications for human health.

## 2 Materials and methods

### 2.1 Intragastric invasion of *Lacticaseibacillus rhamnosus* LRa05

*Lacticaseibacillus rhamnosus* LRa05 (purchased from Wecare Probiotics Co., Ltd.) was cultured in De Man Rogosa Sharpe (MRS) medium (Qingdao Hi-Tech Industrial Park Haibo Biotechnology Co., Ltd. Qingdao, China) and harvested after 18 h of culture at 37°C. A suspension of LRa05 was then obtained by suspending it in sterile water, and the concentration was adjusted to 5 × 10^9^ CFU/mL. Every day, we administered 200 μL of LRa05 suspension by gavage to the mice in the LRa05 group to investigate the effect of LRa05 on chronic low-grade inflammation.

### 2.2 Experimental animals and groups and establishment of the DSS-induced model for chronic low-grade inflammation

We obtained 30 specific pathogen-free male C57BL/6 mice (6–8 weeks old) and obtained permission from the Shanghai Laboratory Animal Center. The study was approved by the Animal Care and Use Committee of the Shanghai Laboratory Animal Center (No.: 2023033012). These mice were kept in an environment with humidity (65% ± 5%), constant temperature (25 ± 2°C), and a 12 h light–dark cycle. In the experiment, we divided the mice into three groups: control (CTL, *n* = 10), dextran sulfate sodium (DSS, MW: 36,000–50,000 Da, YEASEN Biotechnology, Shanghai, China) at low concentration (DSS, *n* = 10) and DSS at low concentration combined with LRa05 (LRa05, *n* = 10).

**Figure 1:**
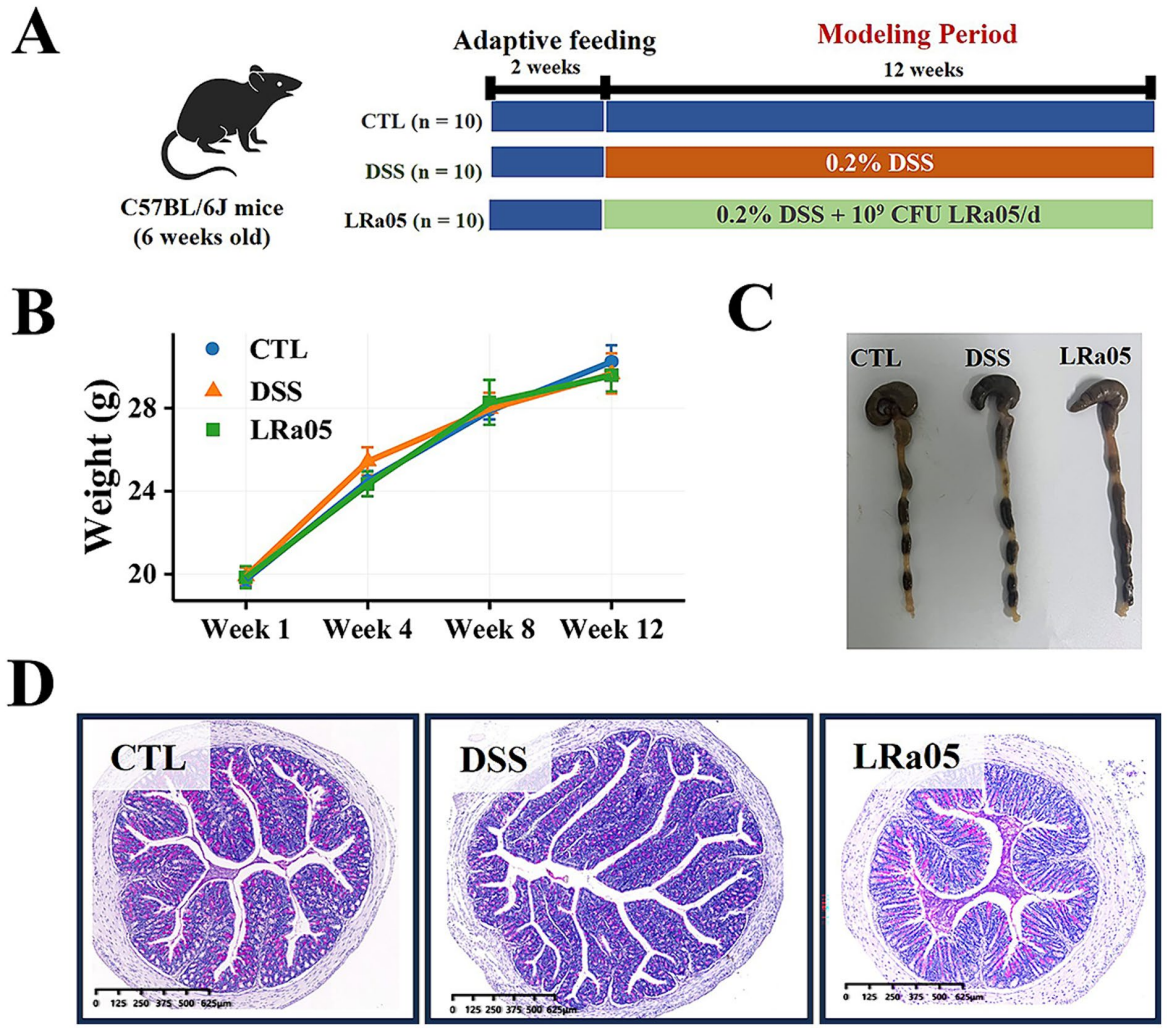
Experimental procedures and the effects of low dextran sulfate sodium (DSS) concentrations on body weight and intestines in mice. **(A)** Experimental procedures. **(B)** Low DSS concentrations have no significant effects on the body weight of mice and the changes in body weight of mice during the experiment. **(C)** Low DSS concentrations have no significant effect on the colon length in mice. The graphical representation of colon length is omitted as statistical analysis revealed no significant differences across treatment groups. **(D)** At the end of the experiment, mice in each group are stained with hematoxylin and eosin (H&E) in the colon. Data are expressed as means ± standard deviations (SDs). CTL, control; LRa05, *Lacticaseibacillus rhamnosus* LRa05.

To establish a model of chronic low-grade inflammation, 0.2% DSS (w/v, g/mL) was dissolved in drinking water and gave it *ad libitum* to the mice in the DSS and LRa05 groups, while the control group drank normal water. The experimental procedure with a 12-week experimental cycle was shown in Figure 1A, and body weight, food intake and health status of the mice were monitored regularly, and blood samples were collected via the orbital vein periodically. At the end of the experiment, the mice were fasted for 12 h and then killed by dislocating the neck. The colon and liver tissues were collected carefully, and fixed in 4% formaldehyde solution until histological analysis.

### 2.3 Determination of serum inflammatory cytokines

The blood samples were centrifuged at 4500 g for 10 min to obtain serum, then stored at −80°C until use. The serum was analyzed for lipopolysaccharides (LPS), and inflammatory cytokines, including, tumor necrosis factor *α* (TNFα), interleukin 4 (IL4), IL6, IL8 and IL10, using enzyme-linked immunosorbent assay (ELISA) kits (Wuhan Chundu Co. Ltd., Wuhan, China), under the guidelines of the manufacturer’s instructions (Funderburg et al., 2013).

### 2.4 Liver function and tissue analysis

Serum alanine aminotransferase (ALT) and aspartate aminotransferase (AST) levels were monitored using an automated biochemical analyzer (Hitachi 7,020, Hitachi, Tokyo, Japan) to assess the liver function of the mice. The colon and liver tissues fixed in formaldehyde solution were dehydrated, embedded with paraffin, cut into 4 μm thick sections and incubated at 37°C for 30 min in the dark (Han et al., 2023). The colon tissue was stained with hematoxylin and eosin (H&E) (Dong et al., 2022), and liver tissue was stained with Oil Red O (Riva et al., 2018), H&E and Masson (Matsumoto et al., 2013) to determine the degree of liver inflammation and fibrosis. In addition, Ethidine fluorescence was observed with 5 μmol/L dihydroethidine (DHE) to detect reactive oxygen species (ROS) in the liver (Chen et al., 2021).

### 2.5 Gut microbiota analysis

We extracted total DNA from mice feces with the QIAamp DNA Stool Mini Kit (Qiagen, Hilden, NRW, Germany). The V3 – V4 region of the 16S rRNA gene was amplified with specific primers (341F: 5 ‘-CCTAYGGGRBGCASCAG-3’, 806R: 5 ‘-GGACTACNGGGTATCTAAT-3’). We then performed double-end sequencing (2 × 300 bp) of the amplicons using the Illumina MiSeq platform. The raw sequence data were analyzed with USEARCH software^1^ to obtain amplicon sequence variants (ASVs) (Edgar, 2013). We assessed alpha diversity using the vegan (Simpson et al., 2022) package, while beta diversity analysis utilized principal coordinate analysis (PCoA) and PERMANOVA significance testing using the adonis2 function of the vegan package. Finally, we used the STAMP software to analyze the differences between groups.

### 2.6 Statistical analysis

The metadata significant differences between the experimental groups were examined using a *t*-test or analysis of variance. For the gut microbiota data, we used a non-parametric test with the Wilcoxon rank sum test to detect significant differences in microbial composition from different groups. All plots were created using the ggplot2 package (Wickham, 2011), correlation analyses were performed using the psych package, and heatmaps were created using the pheatmap package. We performed the analyses under the R language environment version 4.3. The significance level is *p* < 0.05.

### 2.7 Data availability and nucleotide sequence accession numbers

The sequence data used in this article were stored in the NCBI database (accession number, SRA: PRJNA*).

## 3 Results

### 3.1 Low concentrations of DSS did not lead to significant changes in body weight and colon length in mice

Normal growth of the mice was observed throughout the experiment and no hematochezia was observed. In addition, the body weights of the mice showed no significant differences among the treatment groups (Figure 1B). Contrary to the effects observed with high concentrations of DSS (2–3.5%), which are known to significantly shorten colon length, the low concentrations used in this study (0.5–1%) did not lead to any notable reductions in colon length (Figure 1C). This observation supports our use of a milder inflammation model and is consistent with the absence of significant statistical variations, as detailed in the figure legend. The results of H&E staining of the colon (Figure 1D) also showed no inflammatory infiltrates in the tissue, indicating that low concentrations of DSS did not cause colonic inflammation.

### 3.2 DSS caused a significant increase in serum LPS levels as well as the pro-inflammatory cytokines

**Figure 2:**
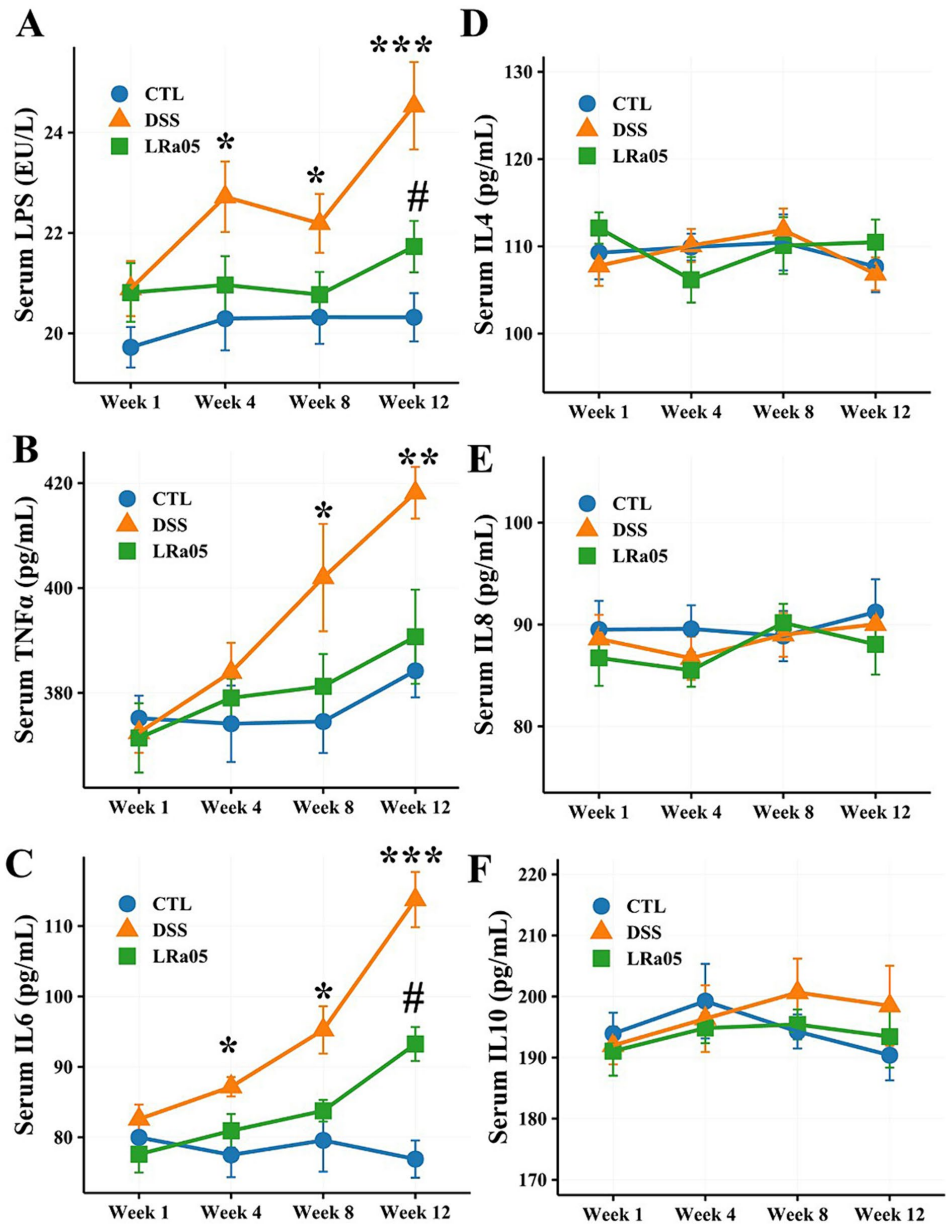
Effect of low DSS concentrations on serum inflammatory cytokine levels in mice. Low DSS concentrations have led to a sustained increase in serum lipopolysaccharide (LPS), tumor necrosis factor α (TNFα), and interleukin 6 (IL6) levels in mice, whereas the LRa05 intervention has significantly decreased serum LPS **(A)**, TNFα **(B)**, and IL6 levels **(C)**. Low DSS concentrations do not lead to significant changes in serum IL4 **(D)**, IL8 **(E)**, and IL10 **(F)** levels in mice. * *p* < 0.05, ** *p* < 0.01 and ***, *p* < 0.001, DSS group compared with CTL group. # *p* < 0.05, LRa05 group compared with CTL group.

According to the results in Figure 2A, serum LPS levels in DSS and LRa05 mice gradually increased with increasing duration of DSS administration. Serum LPS was significantly increased from week 4 in DSS mice compared to CTL mice. However, intervention with LRa05 led to a significant diminution in serum LPS levels, and it was not until week 12 that serum LPS was significantly higher in mice in the LRa05 group than in the CTL group. Of note, mice in the LRa05 group had significantly lower serum LPS levels at week 12 than mice in the DSS group. Similar to LPS, serum TNFα gradually increased in DSS and LRa05 mice with increasing duration of DSS administration (Figure 2B). At week 8, serum TNFα was significantly higher in the DSS group than that in the CTL group. However, intervention with LRa05 significantly reduced serum TNFα levels in the mice, which were not significantly different from the CTL group throughout the experiment, although serum TNFα levels were slightly higher in the mice in the LRa05 group compared with the CTL group. Serum IL6 levels in DSS and LRa05 mice showed a similar increasing trend as TNFα (Figure 2C). Serum IL6 levels were significantly increased from week 4 in DSS mice compared to CTL mice. Intervention with LRa05 significantly reduced serum IL6 levels, especially after week 8, which was significantly lower in the LRa05 group compared to the DSS group. Remarkably, no significant differences in serum IL4 (Figure 2D), IL8 (Figure 2E) and IL10 (Figure 2F) of the mice were observed between the different treatment groups throughout the experimental period.

### 3.3 Protective effect of LRa05 on DSS-induced liver dysfunction in mice

**Figure 3:**
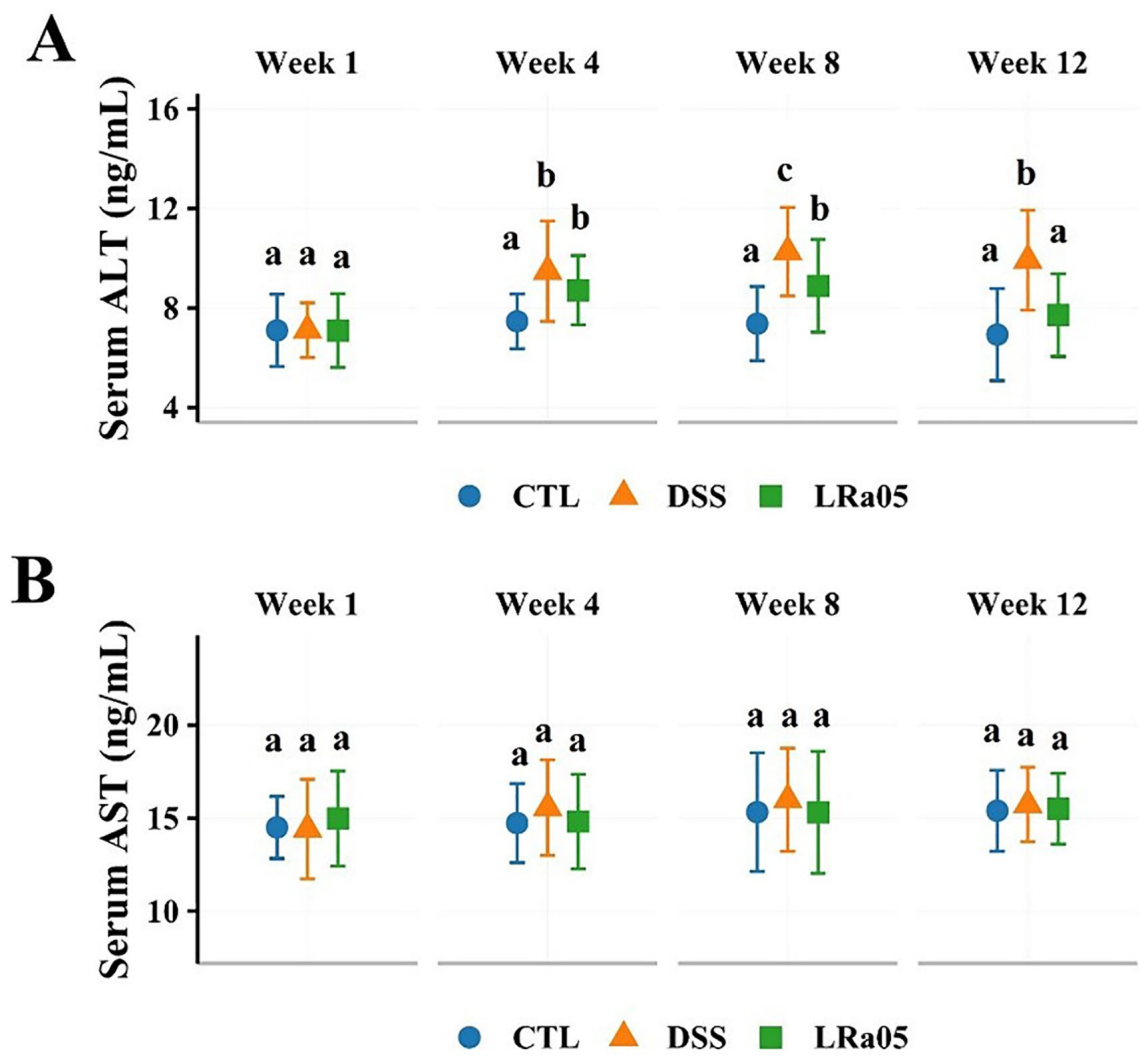
Effect of dextran sulfate sodium (DSS) on liver function in mice. Low concentrations of DSS resulted in a sustained increase in serum alanine aminotransferase (ALT) in mice, and intervention with *Lacticaseibacillus rhamnosus* LRa05 significantly lowered serum ALT levels **(A)**. Low concentrations of DSS did not result in significant changes in serum aspartate aminotransferase (AST) levels in mice **(B)**. Data are expressed as means ± standard deviations (SDs). Different letters in the bar graphs indicate significant differences between the groups. CTL, control; LRa05, *Lacticaseibacillus rhamnosus* LRa05.

Considering that LPS could enter the liver from the intestine via the portal vein, we examined serum ALT and AST levels to assess the effect of DSS on the liver of mice. As shown in Figure 3A, serum ALT levels in DSS mice increased with increasing treatment duration. Intervention with LRa05 significantly inhibited serum ALT levels in the mice, and we observed that at week 8, serum ALT levels in LRa05-treated mice were significantly lower than those in DSS-treated mice and decreased to levels similar to those in CTL mice at week 12. In contrast to ALT, although fluctuations in serum AST levels were observed in the mice throughout the experiment, there were no significant differences between groups (Figure 3B). These results suggested that sustained low concentrations of DSS resulted in impaired liver function in mice, whereas LRa05 protected the liver.

**Figure 4:**
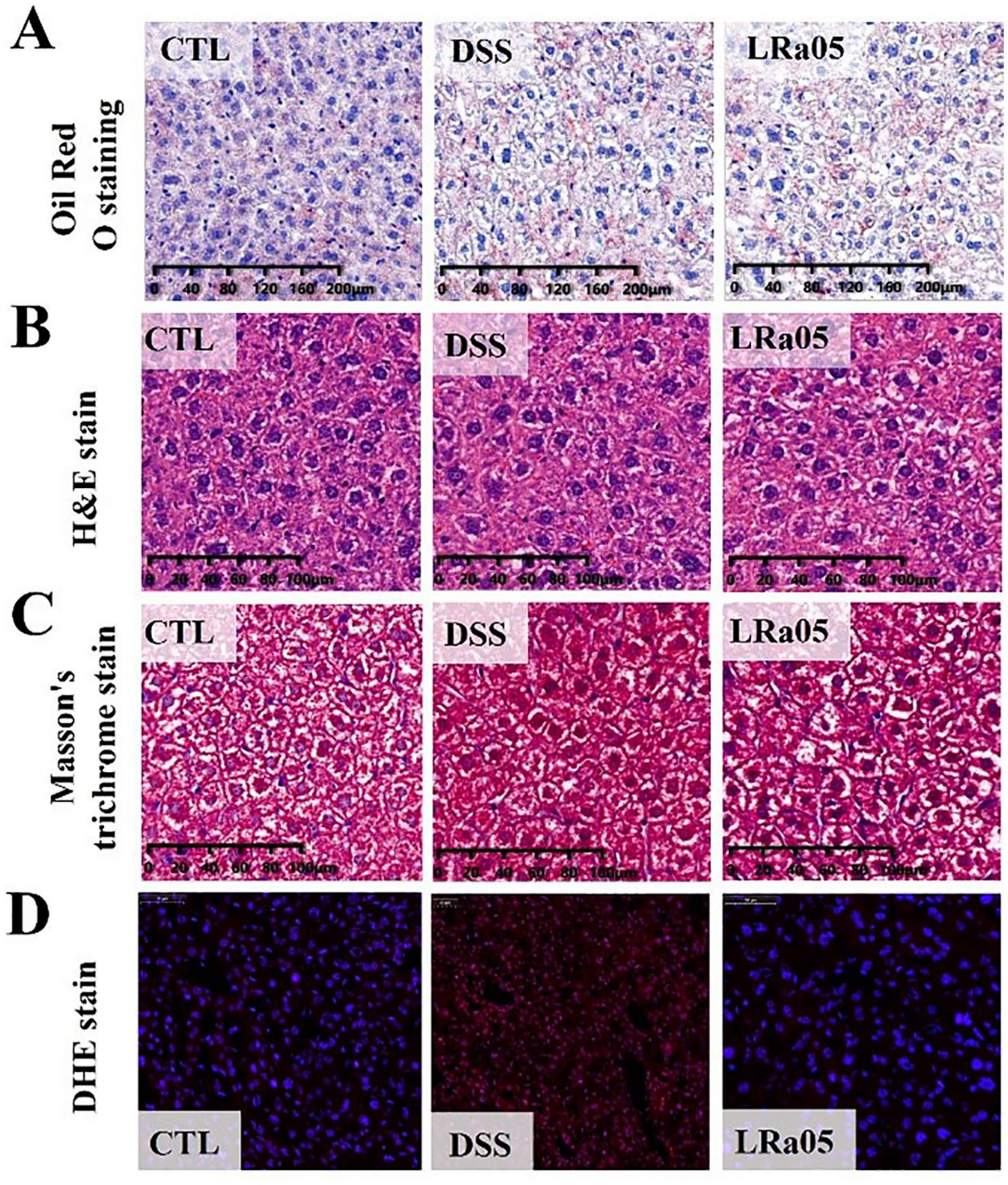
Mouse liver tissue damage caused by DSS and the protective effect of *Lacticaseibacillus rhamnosus* LRa05 on mouse liver tissues. **(A)** Red Oil O staining of the liver showing no fat accumulation in the liver. **(B)** H&E staining of the liver showing no fine hepatocellular necrosis and inflammatory reactions. **(C)** Masson staining of the liver showing no fibrosis of the liver tissue. **(D)** DSS has increased reactive oxygen species (ROS) levels in the liver tissue of mice, and LRa05 has significantly reduced ROS levels in the liver. CTL, control; LRa05, *Lacticaseibacillus rhamnosus* LRa05.

To gain a better insight into the effects of low concentrations of DSS on liver tissue, we performed tissue staining analysis. As shown in Figure 4A, low concentrations of DSS for 12 weeks did not lead to the accumulation of liver fat in mice. In addition, the results of hepatic H&E staining showed that DSS did not cause inflammatory infiltration and cell necrosis in the liver of mice (Figure 4B). In the results of Masson staining, we could not observe fibrosis in the liver of mice, and there was no significant difference between the groups of mice (Figure 4C). Notably, the results of liver DHE showed that sustained low concentrations of DSS led to a significant increase in reactive oxygen species in the liver of mice, and the intervention with LRa05 significantly reduced the level of reactive oxygen species in liver tissue compared with the DSS group (Figure 4D).

### 3.4 LRa05 alleviates disturbances of the gut microbiota induced by prolonged low concentrations of DSS in mice

**Figure 5:**
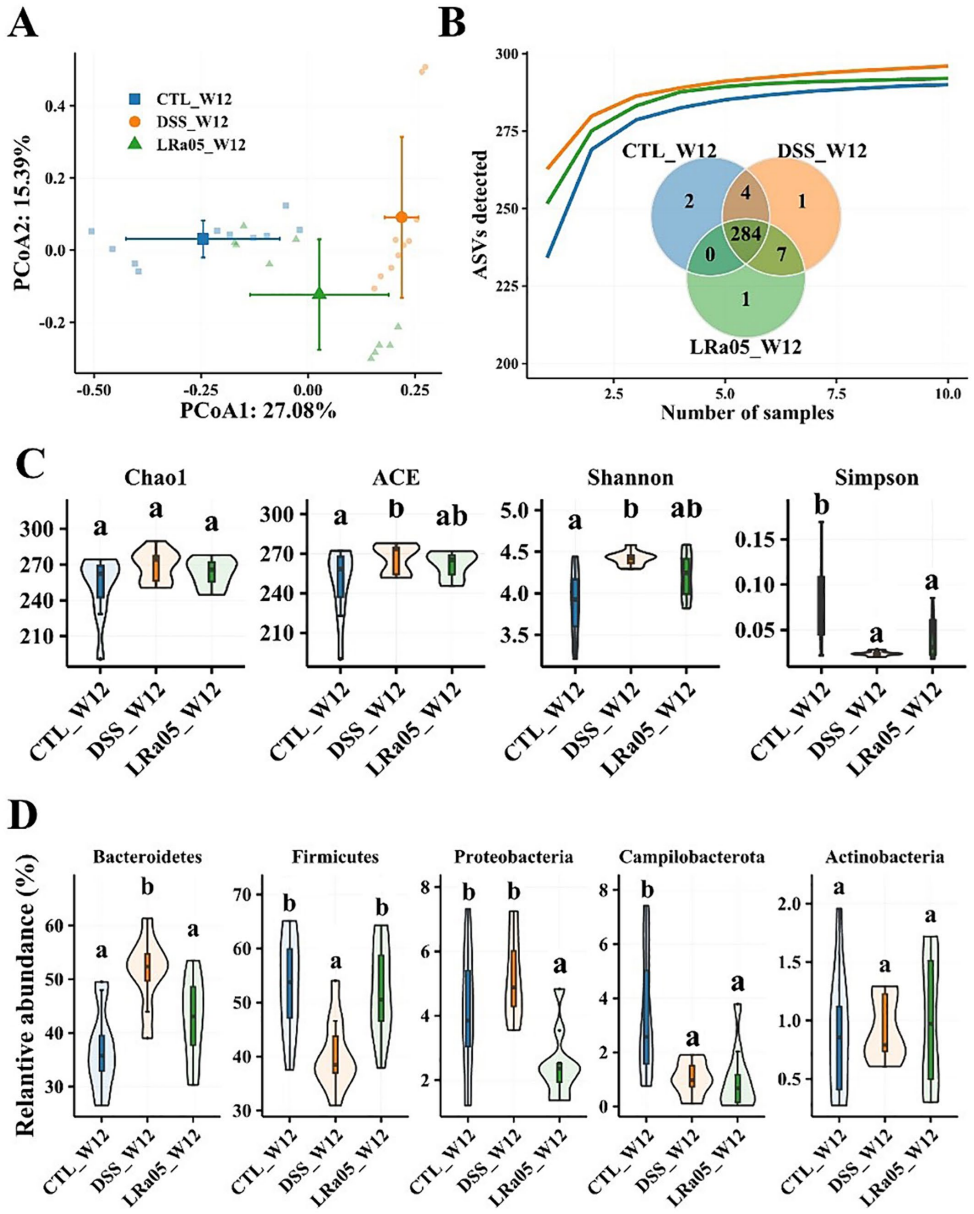
Effect of 0.2% DSS on the intestinal flora of mice and improvement of intestinal flora by *Lacticaseibacillus rhamnosus* LRa05 intervention. **(A)** Principal coordinate analysis of the gut microbiota in different groups of mice; DSS has caused significant changes in the gut microbiota. **(B)** Species accumulation curves of gut microbiota in different groups of mice; Venn plots show differences in gut microbiota between different groups. **(C)** Effect of DSS and LRa05 on the alpha diversity of the gut microbiota of mice. **(D)** Effect of DSS and LRa05 intervention on the graduation of the gut microbiota of mice at the phylum level. Different letters in the violin plots indicate significant differences between the groups. CTL, control; LRa05, *Lacticaseibacillus rhamnosus* LRa05. Different letters above the data bars or points indicate statistically significant differences between groups, while identical letters indicate no significant differences as determined by post-hoc tests.

The results of the PCoA analysis showed (Figure 5A; Supplementary Table S1) that DSS caused significant changes in the beta diversity of the gut microbiota of mice, while the intervention with LRa05 significantly modulated the gut microbiota. The results of species accumulation curves showed that 284 ASVs were present in the gut microbiota of mice in the three groups, and 2, 1 and 1 unique ASVs were present in the CTL, DSS and LRa05 groups, respectively (Figure 5B). This indicated that DSS did not cause significant changes in the types of gut microbiota, while the main difference was manifested in the relative abundance of the microbiota. This was also confirmed by the analysis of alpha diversity, as shown in Figure 5C, where the intervention with DSS resulted in a significant increase in the richness (ACE index) and diversity (Shannon index) of the mice gut microbiota. However, the results of Chao1, ACE and Shannon indices showed that LRa05 was able to significantly decrease the DSS-induced changes in the alpha diversity of the gut microbiota.

We also analyzed the differences in the abundance of the gut microbiota in each group of mice at the phylum level (Figure 5D). In each group of mice, the gut microbiota consisted mainly of *Bacteroidetes*, *Firmicutes*, *Proteobacteria*, *Campilobacterota* and *Actinobacteria*, which accounted for more than 99%. The use of DSS resulted in a significant increase in the relative abundance of *Bacteroidetes* in the gut microbiota of mice, while *Firmicutes* and *Campilobacterota* showed a significant decrease in relative abundance. The relative abundance of *Actinobacteria* did not differ significantly among the three groups. The intervention with LRa05 reversed the DSS-induced changes in the gut microbiota to some extent, as shown by the increase in the relative abundance of the *Firmicutes* and the decrease in the relative abundance of *Bacteroidetes*. In addition, interestingly, the relative abundance of Proteobacteria in the gut microbiota of mice in the LRa05 group was significantly reduced compared to the CTL and DSS groups.

**Figure 6:**
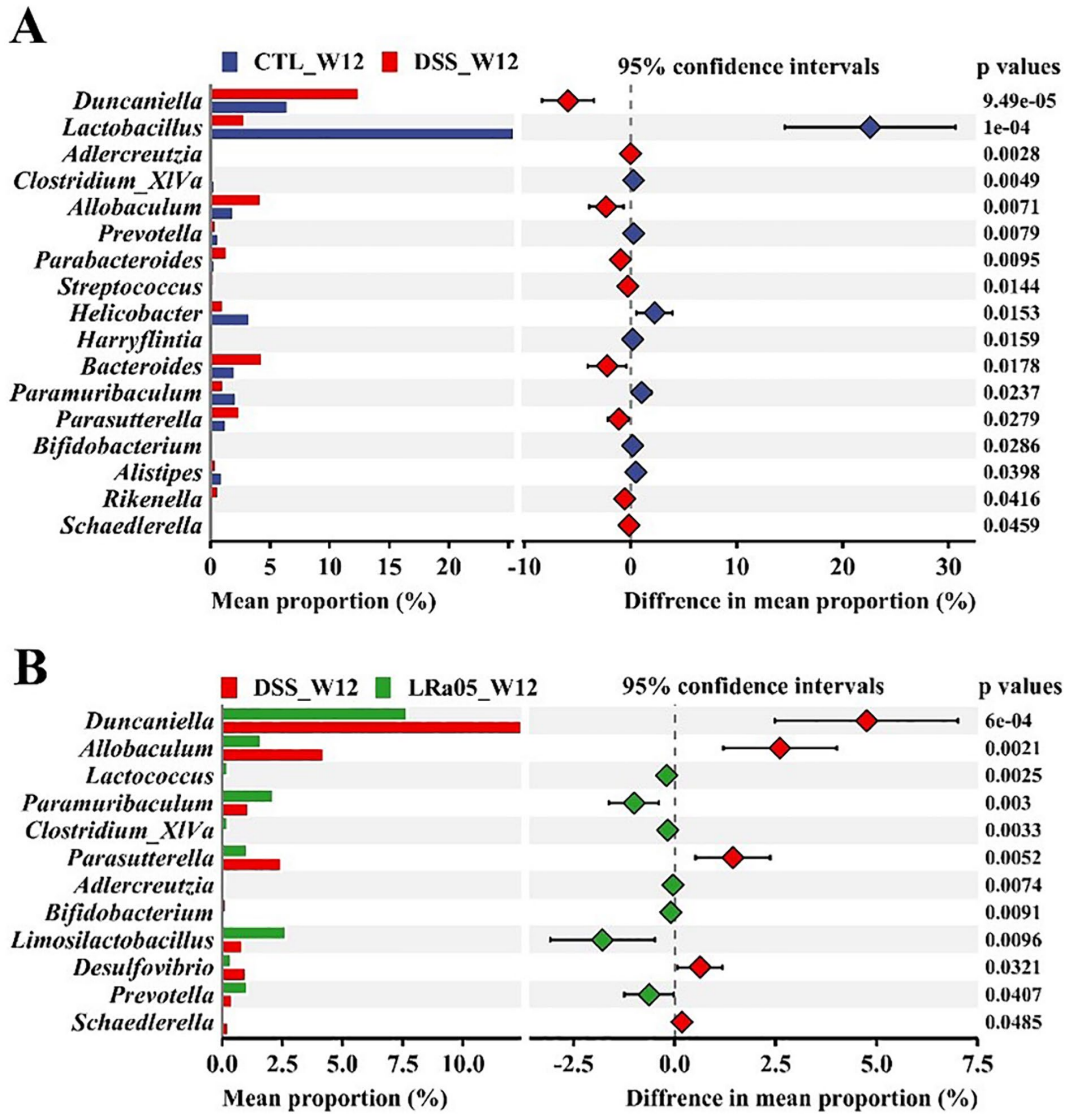
Investigation of the effects of DSS and *Lacticaseibacillus rhamnosus* LRa05 on the intestinal microbiota of mice at the genus level. Low DSS concentrations have led to a decrease in beneficial gut bacteria and an increase in harmful bacteria in mice **(A)**, whereas the intervention with LRa05 shows an ameliorative effect on gut microbiota **(B)**. CTL, control; LRa05, *Lacticaseibacillus rhamnosus* LRa05.

The differences in the gut microbiota of mice from each group at the end of the experiment were analyzed at the genus level. Compared to the CTL group, treatment with low concentrations of DSS for 12 weeks resulted in a significant decrease in the relative abundance of the beneficial bacteria *Lactobacillus*, *Clostridium_XlVa* and *Bifidobacterium*, and the pathogenic bacteria *Duncaniella*, *Allobaculum*, *Parabacteroides* and *Parasutterella* in the gut of the mice (Figure 6A). Treatment with LRa05 reversed this trend and significantly improved the DSS-induced changes in the gut microbiota. Compared to the DSS group, intervention with LRa05 significantly decreased the relative abundance of *Duncaniella*, *Allobaculum* and *Parasutterella*, and increased the relative abundance of beneficial bacteria such as *Lactobacillus*, *Clostridium_XlVa*, *Bifidobacterium* and *Limosilactobacillus* (Figure 6B).

### 3.5 DSS-induced dynamic changes in the gut microbiota and the sustained improvement of the gut microbiota by LRa05

**Figure 7:**
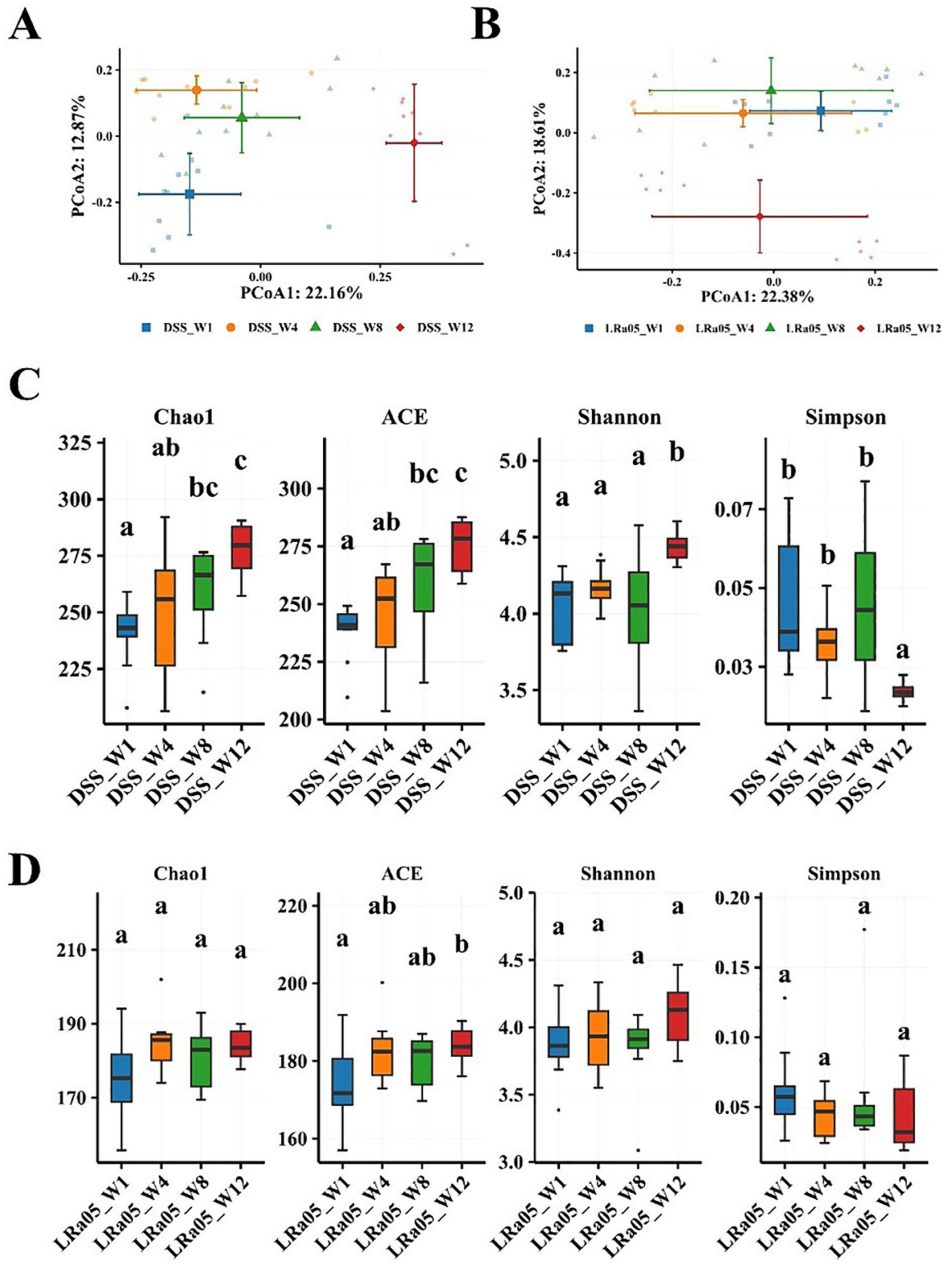
Changes in the intestinal flora of mice at different time points in the DSS and *Lacticaseibacillus rhamnosus* LRa05 groups. At different time points, a significant difference in the beta diversity of the intestinal microbiota is noted between mice treated with DSS **(A)** and LRa05 **(B)**. Differences in the alpha diversity of gut microbiota between mice treated with DSS **(C)** and LRa05 **(D)** at different time points. Different letters in the boxplots indicate significant differences between the groups. Different letters above the data bars or points indicate statistically significant differences between groups, while identical letters indicate no significant differences as determined by post-hoc tests.

To further analyze the effect of DSS on the gut microbiota of mice, we investigated the changes in the gut microbiota of mice over time. We collected feces from DSS and LRa05 mice after 1, 4, 8 and 12 weeks of intervention and performed gut microbiota analysis. As shown in the results of the PCoA analysis, the beta diversity of the gut microbiota of DSS mice (Figure 7A; Supplementary Table S2) and LRa05 mice (Figure 7B; Supplementary Table S3) differed significantly at different time points. In addition, the beta gut microbiota was observed to be more distant in DSS mice compared to LRa05 mice at different time points. This suggested that DSS led to disruption of the gut microbiota, while intervention with LRa05 can effectively reduce the disruption of the gut microbiota and reduce the differences in the gut microbiota at different time points. Similar to beta diversity, alpha diversity also changed over time. The abundance (Chao1 and ACE index) and diversity (Shannon index) of the gut microbiota gradually increased in the DSS mice, especially the alpha diversity of the gut microbiota increased significantly at week 12 compared to week 1 (Figure 7C). In contrast to the DSS group, the richness and diversity of the gut microbiota of the mice in the LRa05 group fluctuated but were relatively stable overall, and only the ACE index changed significantly (Figure 7D).

As shown by the analysis of the species accumulation curve (Supplementary Figure S1), no significant new bacteria emerged in the gut microbiota of DSS and LRa05 mice over time. These results suggested that the differences in the gut microbiota of mice were mainly associated with changes in the relative abundance of the gut microbiota. DSS led to a disruption of the gut microbiota, whereas LRa05 can effectively reduce the DSS-induced changes in the gut microbiota. At the phylum level, the gut microbiota was mainly composed of *Bacteroidetes*, *Firmicutes*, *Proteobacteria*, *Campilobacterota* and *Actinobacteria* (Supplementary Figures S2A,B). We observed DSS-induced fluctuations in the abundance of the gut microbiota at the phyla level, which was mainly manifested by a decrease in the relative abundance of *Firmicutes* and an increase in the relative abundance of *Bacteroidetes* and *Proteobacteria* (Supplementary Figure S2C). However, the intervention of LRa05 was able to effectively reduce the changes in the gut microbiota caused by DSS (Supplementary Figure S2D).

### 3.6 Correlation analysis and critical organism genus over time

**Figure 8:**
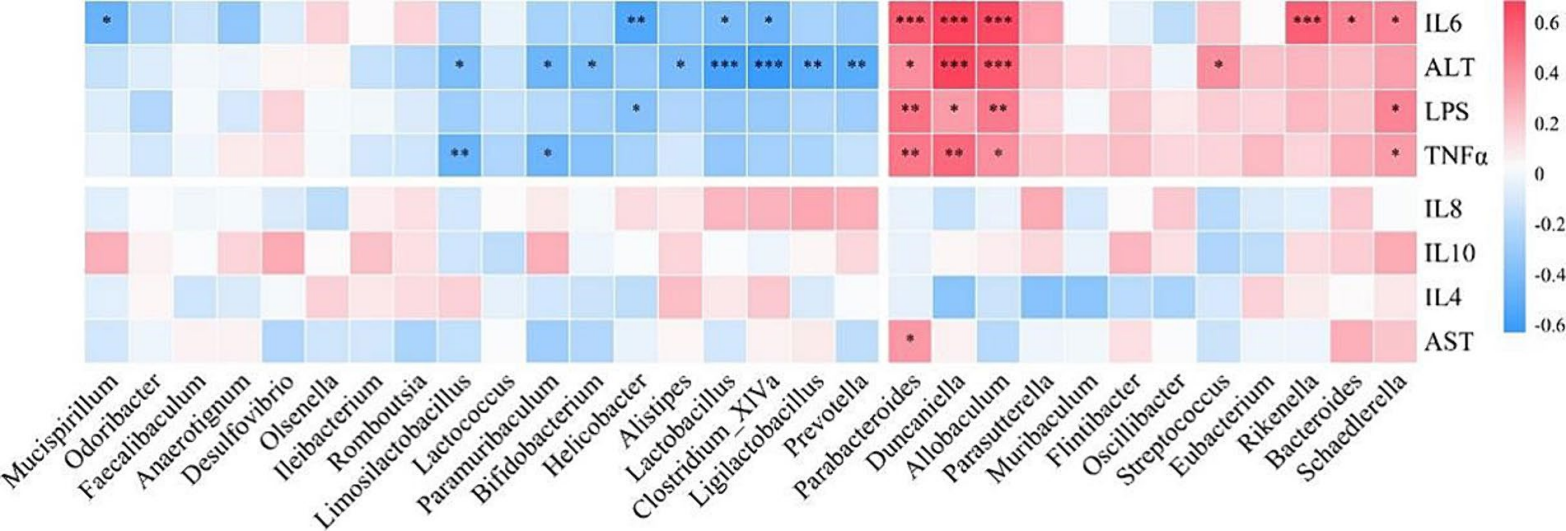
Correlation analysis of intestinal flora with inflammatory cytokines, serum alanine aminotransferase (ALT), and aspartate aminotransferase (AST). TNFα, tumor necrosis factor α; interleukin, IL.

To investigate the relationship between the gut microbiota and inflammatory cytokines and liver parameters, we performed a Spearman correlation analysis (Figure 8). We found that microbial genera enriched in the gut of DSS mice, such as *Duncaniella*, *Allobaculum* and *Parabacteroides*, were positively correlated with LPS, TNFα, IL6 and ALT. However, microbial genera that decreased in DSS mice, such as *Clostridium_XlVa*, *Limosilactobacillus*, *Lactobacillus* and *Bifidobacterium*, were negatively correlated with LPS, TNFα, IL6 and ALT.

**Figure 9:**
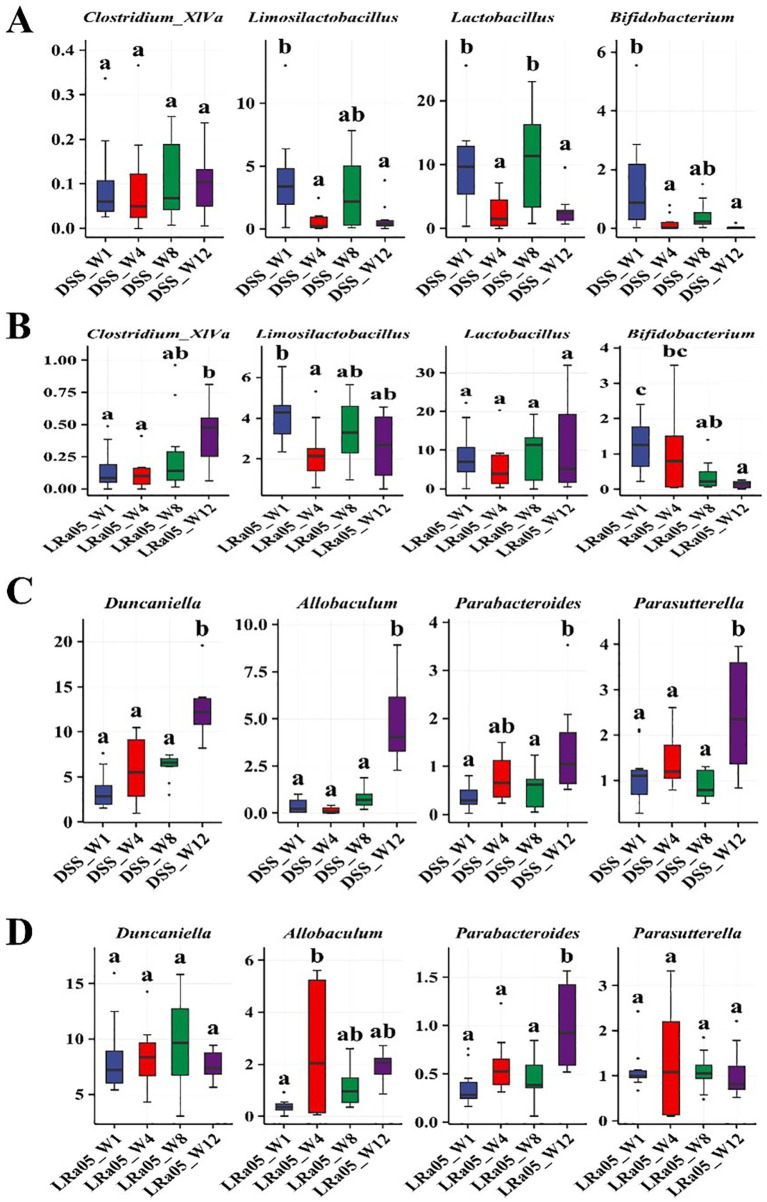
Relative abundance of different bacteria over time in different groups of mice. Changes in the abundances of beneficial bacteria *Clostridium_XlVa*, *Limosilactobacillus*, *Lactobacillus*, and Bifidobacterium at different time points in the DSS group **(A)** and *Lacticaseibacillus rhamnosus* LRa05 groups **(B)**. Changes in the abundances of opportunistic pathogens *Duncaniella*, *Allobaculum*, Parabacteroides, and *Parasutterella* at different time points in the DSS **(C)** and LRa05 groups **(D)**. Different letters in the boxplot indicate significant differences between the groups. Different letters above the data bars or points indicate statistically significant differences between groups, while identical letters indicate no significant differences as determined by post-hoc tests.

We further investigated the evolution of these microbial genera over time. We observed no significant change in the relative abundance of the beneficial bacterium *Clostridium_XlVa* over time in the DSS group (Figure 9A), but the intervention with LRa05 significantly increased the relative abundance of *Clostridium_XlVa* (Figure 9B). In addition, the relative abundance of *Limosilactobacillus*, *Lactobacillus* and *Bifidobacterium* in the DSS group decreased significantly over time (Figure 9A), while the intervention with LRa05 significantly increased the relative abundance of *Limosilactobacillus* and *Lactobacillus* (Figure 9B). We observed that the relative abundances of the opportunistic pathogens *Duncaniella*, *Allobaculum*, *Parabacteroides* and *Parasutterella* gradually increased over time in the DSS group (Figure 9C). In particular, the relative abundance of these microbial genera increased significantly at week 12 compared to week 1 (Figure 9C). While the relative abundances of *Duncaniella*, *Allobaculum*, and *Parasutterella* initially increased and then decreased after treatment with LRa05, the relative abundances of these microbial genera eventually remained stable (Figure 9D).

## 4 Discussion

In this study, we investigated in detail the chronic inflammation induced by low concentrations of DSS and the therapeutic effect of the probiotic *Lacticaseibacillus rhamnosus* LRa05. The model induced by DSS mimicked the mild inflammation observed in patients with IBD and its effects on liver function (Perse and Cerar, 2012). By analyzing systemic inflammatory markers, liver function and gut microbiota, we were able to uncover a number of changes attributable to DSS and speculate on the therapeutic mechanism of LRa05. This approach allowed us to investigate subtle and influential differences in inflammation. The results showed that DSS at low concentration (0.2%) effectively induced chronic low-grade inflammation in mice without significantly altering body weight and colon length.

Our study on chronic low-grade inflammation caused by low concentrations of DSS and the intervention of *Lacticaseibacillus rhamnosus* LRa05 has provided some crucial insights. We found that low concentrations of DSS did not induce colonic inflammation and significant weight changes in mice, which was in contrast to the intense inflammation observed in the high concentration DSS model (Dong et al., 2022). However, the increased systemic inflammatory response, particularly the significant increase in serum levels of LPS, TNFα and IL6, indicated that chronic inflammation was present in the DSS model. LPS can induce an increase in the production of inflammatory cytokines through the NF-κB signal pathways (Matsuguchi et al., 2000). As a major pro-inflammatory cytokine, TNFα contributes to cell death, triggers T cells and macrophages, and draws in concentrated granulocytes to worsen inflammatory infiltration. IL-6 can stimulate colon cancer and activate the NF-κB pathway, as well as regulate intestinal tight junction, induce intestinal immunity, and incite intestinal inflammation (Cuellar-Nunez et al., 2021). The present study is consistent with the increase in serum inflammatory markers observed in patients with IBD (Szymanska et al., 2023). It has been shown that no colitis-related symptoms were observed in mice fed only a fiber-free diet, but these animals are very sensitive to low-dose (0.5%) dextran sulfate sodium (DSS)-induced colitis models (Shen et al., 2021). Intervention with LRa05 suppressed the increase in these systemic inflammatory markers to some extent, indicating its role in combating inflammation.

Our analysis revealed that prolonged treatment with low concentrations of DSS led to increased ALT levels in the serum of mice, signaling liver damage. Elevated ALT is a well-recognized biomarker of liver cell injury and its rise in our model mirrors the liver dysfunction commonly observed in patients with inflammatory bowel disease (IBD), reinforcing the clinical relevance of our findings (Hanafy et al., 2016; Mendes et al., 2007). The correlation between IBD and liver disease is well-documented, and our results further underscore this link, highlighting the potential systemic impact of localized intestinal inflammation on liver health. Furthermore, intervention with *Lacticaseibacillus rhamnosus* LRa05 led to a significant reduction in ALT levels. This observation aligns with other studies demonstrating the hepatoprotective effects of probiotics, suggesting that LRa05 may mitigate liver damage through mechanisms possibly involving anti-inflammatory actions and enhancement of intestinal barrier integrity (Mendes et al., 2007). This protective effect is crucial, as it suggests that modulation of the gut microbiota by probiotics can extend beyond the gut to influence systemic organ functions, including the liver. Additionally, despite the absence of overt histopathological changes in liver tissues upon DSS administration, we noted a significant increase in ROS levels. This increase in ROS indicates oxidative stress, which is a known mediator of cellular damage and a contributor to the pathogenesis of liver diseases (Cichoż-Lach and Michalak, 2014). The oxidative stress observed further supports the systemic nature of the inflammatory response induced by DSS, and underscores the importance of antioxidant therapies in managing such conditions. The dual role of LRa05 in reducing serum ALT levels and oxidative stress highlights its therapeutic potential not only in managing gut inflammation but also in protecting against liver damage. Interestingly, while ALT levels showed significant elevation, the AST levels did not exhibit the same trend. We hypothesize that this lack of significant AST elevation may be due to the specific characteristics of DSS-induced liver injury. DSS might preferentially affect certain hepatocyte populations or metabolic pathways, thereby limiting AST release into the serum. Moreover, liver damage in this model may not have progressed to the point of widespread hepatocellular necrosis, which is typically associated with a marked increase in AST. The differential changes in ALT and AST further suggest that the liver injury in this context may be more localized or specific in nature.

The changes in gut microbiota composition induced by DSS and the subsequent restoration of the intervention with LRa05 reveal complex interactions between the microbiome and host health. DSS leads to dysbiosis of the gut microbiota, which is usually manifested by alterations in the composition and diversity of the microbiota (Xu et al., 2021). Our results showed that DSS treatment led to a decrease in the relative abundance of beneficial bacteria and an increase in the proliferation of potentially harmful bacteria, while the intervention with LRa05 helped to maintain the balance of microbial communities in the gut. In a recent study, the novel gut pathogen *Duncaniella muricolitica* was identified and isolated by microbiome analysis, machine learning and targeted anaerobic culture, demonstrating that it plays a dominant role in the DSS model (Forster et al., 2022). In this study, we observed a significant increase in the relative abundance of the genus *Duncaniella* in the DSS group, while the LRa05 intervention effectively suppressed the increase in the abundance of *Duncaniella* (Figure 9). In addition, *Allobaculum* was reported to be involved in the regulation of ANGPTLT4 expression in the intestine of mice fed a high-fat diet. The relative abundance of *Allobaculum* in the ileum and colon was significantly higher in mice fed a high-fat diet than in controls. Treatment with a high-fat diet significantly increased the expression of ANGPTL4 in the ileum and colon of mice, and the expression of ANGPTL4 was positively correlated with the relative abundance of *Allobaculum* (Zheng et al., 2021). *Allobaculum* was also found enriched in the intestine of DSS-induced colitis mice (Chen et al., 2019). In this study, we observed a significant increase in the relative abundance of *Allobaculum* genera in the DSS group, while the LRa05 intervention brought the abundance of *Allobaculum* genera to a stable level (Figure 9). *Limosilactobacillus* and *Lactobacillus* are common probiotics, which have been proven to be favorable factors in liver protection (Ding et al., 2022).

In the correlation analysis, we found that microbial genera enriched in the gut of DSS mice were positively correlated with LPS, TNFα, IL6 and ALT, while beneficial microbes were negatively correlated. The persistent increase in serum LPS, TNFα and IL6 levels in the DSS group emphasized the systemic effects of chronic low-grade inflammation. The intervention with LRa05 proved critical in attenuating this impairment, particularly in the later phase of the study where levels of these markers were significantly reduced. Elevated ALT levels in the DSS group indicate liver impairment, and the intervention with LRa05 played a key role in alleviating this impairment. Histologic analysis provided further evidence that LRa05 exerted a protective effect in attenuating DSS-induced oxidative stress in the liver. LRa05 promotes the growth of beneficial bacteria by regulating the gut microbiota while inhibiting potentially pathogenic strains. The resulting increased microbial diversity and stability contributed to the observed anti-inflammatory effects and liver protection.

**Figure 10:**
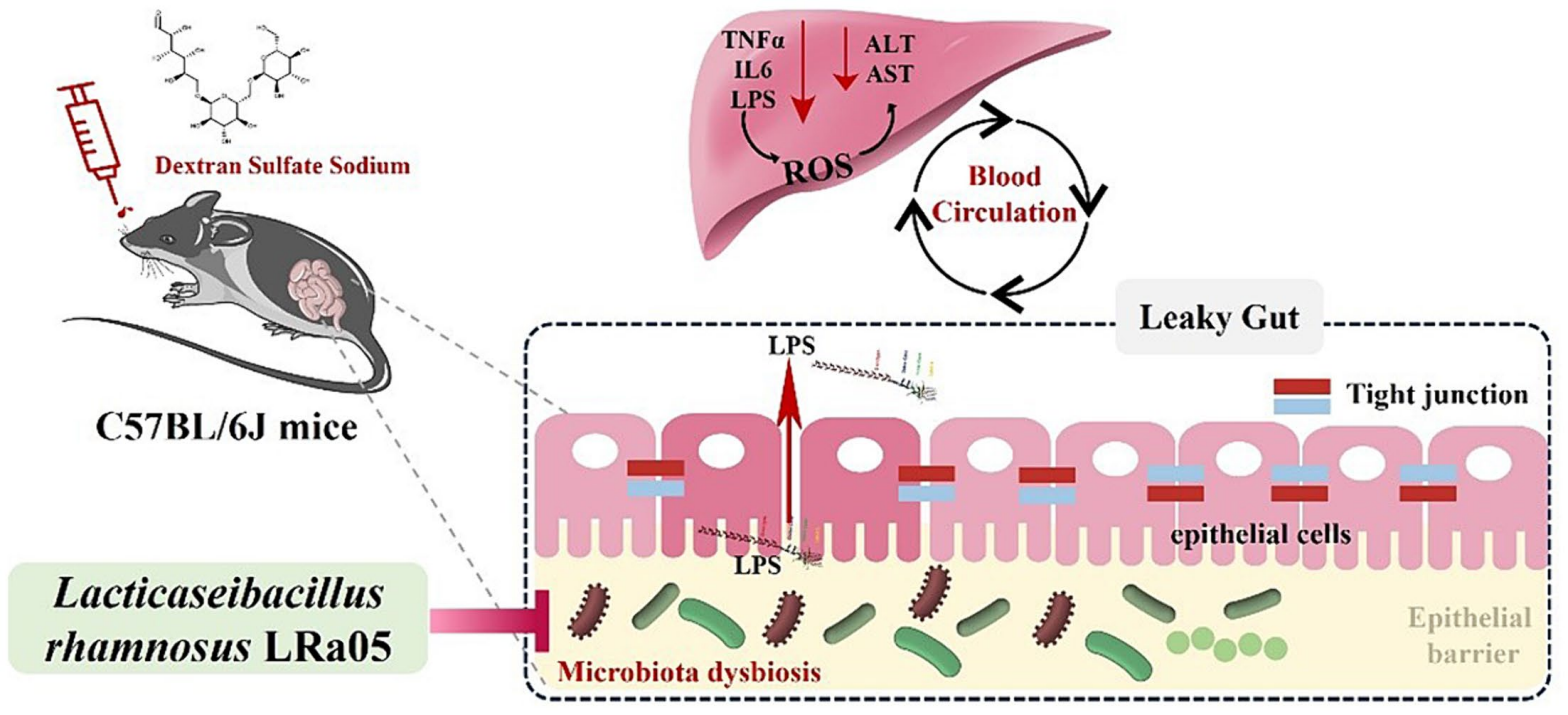
Speculated mechanisms by which *Lacticaseibacillus rhamnosus* LRa05 alleviates the inflammation and liver injury induced by low concentrations of dextran sulfate sodium in mice.

The intervention time of LRa05 was dynamic and its protective effect gradually became apparent within 12 weeks of the experimental cycle. This suggested the need to consider the timing of administration when assessing the effects of probiotic therapy. Based on the above results, we hypothesize the mechanism of action of LRa05: chronic low concentrations of DSS lead to disturbances in the gut microbiota of mice, a decrease in the relative abundance of beneficial bacteria and an increase in the relative abundance of opportunistic pathogens. This disruption of the gut microbiota can cause microenteric fistulas that lead to increased LPS concentrations in the circulatory system, triggering an inflammatory response. Disturbed gut microbiota and increased LPS concentrations also trigger hepatic oxidative stress and ultimately impair liver function. The intervention of LRa05 was able to improve the gut microbiota, reverse DSS-induced disruption of the gut microbiota in mice, increase the relative abundance of beneficial bacteria and inhibit the excessive proliferation of opportunistic pathogens, thereby reducing the LPS content in the circulatory system. The improvement of gut microbiota and blood LPS levels could further reduce the inflammatory response and protect the liver from oxidation-induced functional damage. The mechanism of action of LRa05 may be to maintain the integrity of the intestinal mucosa by regulating the balance of the gut microbiota and reducing the penetration of LPS into the gut. This inhibits inflammatory reactions and reduces oxidative stress in the liver, which ultimately has a protective effect (Figure 10).

Our study highlights the characteristics of chronic inflammation in a low concentration DSS model and the therapeutic potential of the probiotic LRa05. Using low-dose DSS allowed us to better mimic the chronic inflammatory state seen in patients, offering more accurate insights into the progression of inflammation, which is a novel approach in IBD research. Through a detailed temporal analysis, we observed significant changes in gut microbiota, systemic inflammatory markers, and liver function, shedding light on the dynamic effects of LRa05 intervention. We also explored correlations between the microbiome, cytokines, and liver function, revealing important interactions that influence disease development. While we demonstrated LRa05’s protective effects, further studies are needed to investigate its molecular mechanisms and immunomodulatory pathways, along with other relevant biomarkers. Overall, our findings offer new perspectives on IBD pathogenesis and probiotic treatment, providing a foundation for future experimental and clinical research.

## Data Availability

The sequence data used in this article were stored in the NCBI database (accession number, PRJNA1045051).

## Footnotes

^1^ https://www.drive5.com/usearch/

## Supplementary material

SUPPLEMENTARY FIGURE S1 Species accumulation curves of the gut microbiota at different time points in dextran sulfate sodium (DSS) **(A)** and *Lacticaseibacillus rhamnosus* LRa05 **(B)** mice.

SUPPLEMENTARY FIGURE S2 Differential analysis of the intestinal microbiota at the phylum level between dextran sulfate sodium (DSS) and *Lacticaseibacillus rhamnosus* LRa05 mice at different time points. Accumulation diagram of intestinal microbiota abundance at the phylum level in DSS **(A)** and LRa05 **(B)** mice. Differences in intestinal microbiota abundance at phylum level between DSS **(C)** and LRa05 **(D)** mice at different time points. Different letters in the violin plots indicate significant differences between the group.

SUPPLEMENTARY TABLE S1 The tests for microbial community composition dissimilarity between pairs of groups were performed using nonparametric permutational multivariate ANOVA with the adonis2 function of vegan in R (version 4.3).

SUPPLEMENTARY TABLE S2 The tests for microbial community composition dissimilarity between pairs of groups were performed using nonparametric permutational multivariate ANOVA with the adonis2 function of vegan in R (version 4.3).

SUPPLEMENTARY TABLE S3 The tests for microbial community composition dissimilarity between pairs of groups were performed using nonparametric permutational multivariate ANOVA with the adonis2 function of vegan in R (version 4.3).
