# Supplementary material for: Lacticaseibacillus rhamnosus LRa05 mediates dynamic regulation of intestinal microbiota in mice with low-dose DSS-induced chronic mild inflammation
Source: Front Microbiol. 2024 Oct 8;15:1483104. doi: 10.3389/fmicb.2024.1483104 (PMC11496787; doi:10.3389/fmicb.2024.1483104)
Supplement: SUPPLEMENTARY FIGURE S1 — Species accumulation curves of the gut microbiota at different time points in dextran sulfate sodium (DSS) (A) and Lacticaseibacillus rhamnosus LRa05 (B) mice. [file Data_Sheet_1.docx]

Supplementary Material

# Supplementary Figures and Tables

## Supplementary Figures


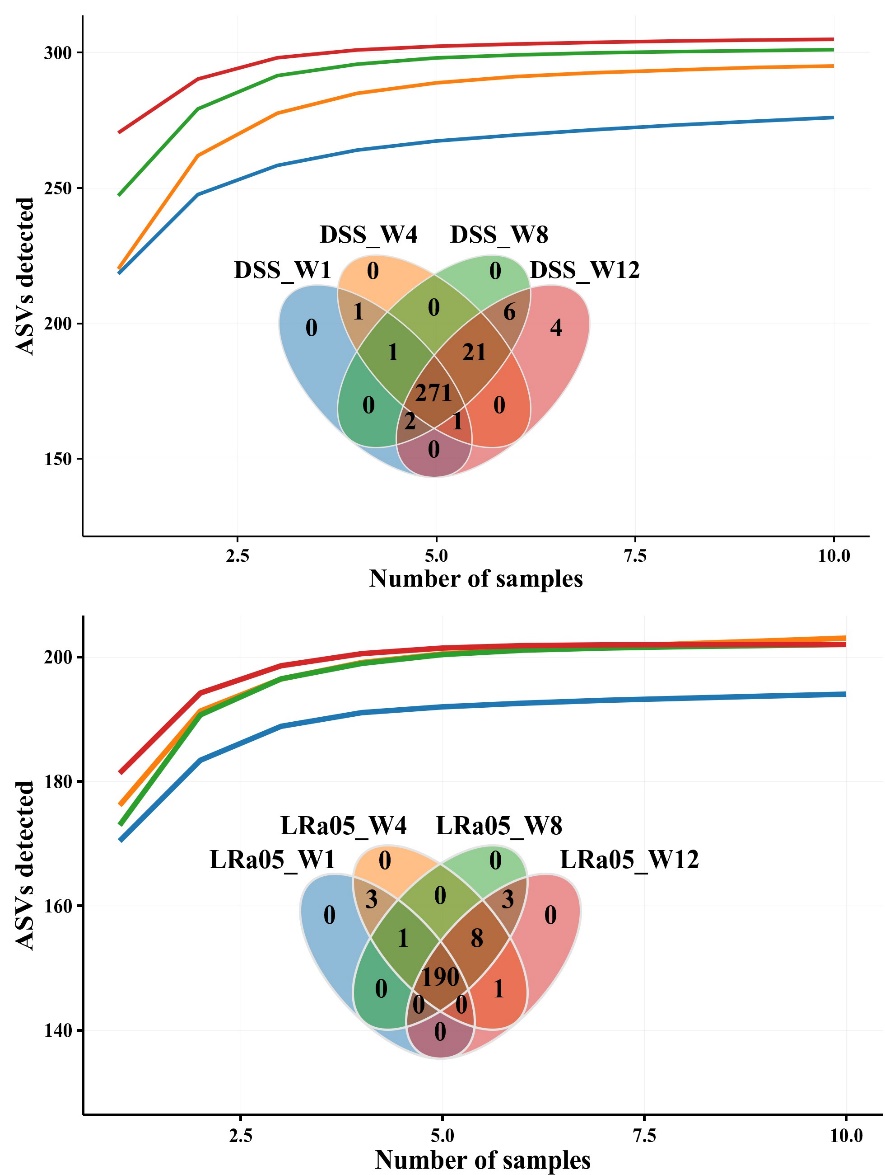


**Supplementary Figure 1.** Species accumulation curves of the gut microbiota at different time points in dextran sulfate sodium (DSS) (A) and *Lacticaseibacillus* *rhamnosus* LRa05 (B) mice.


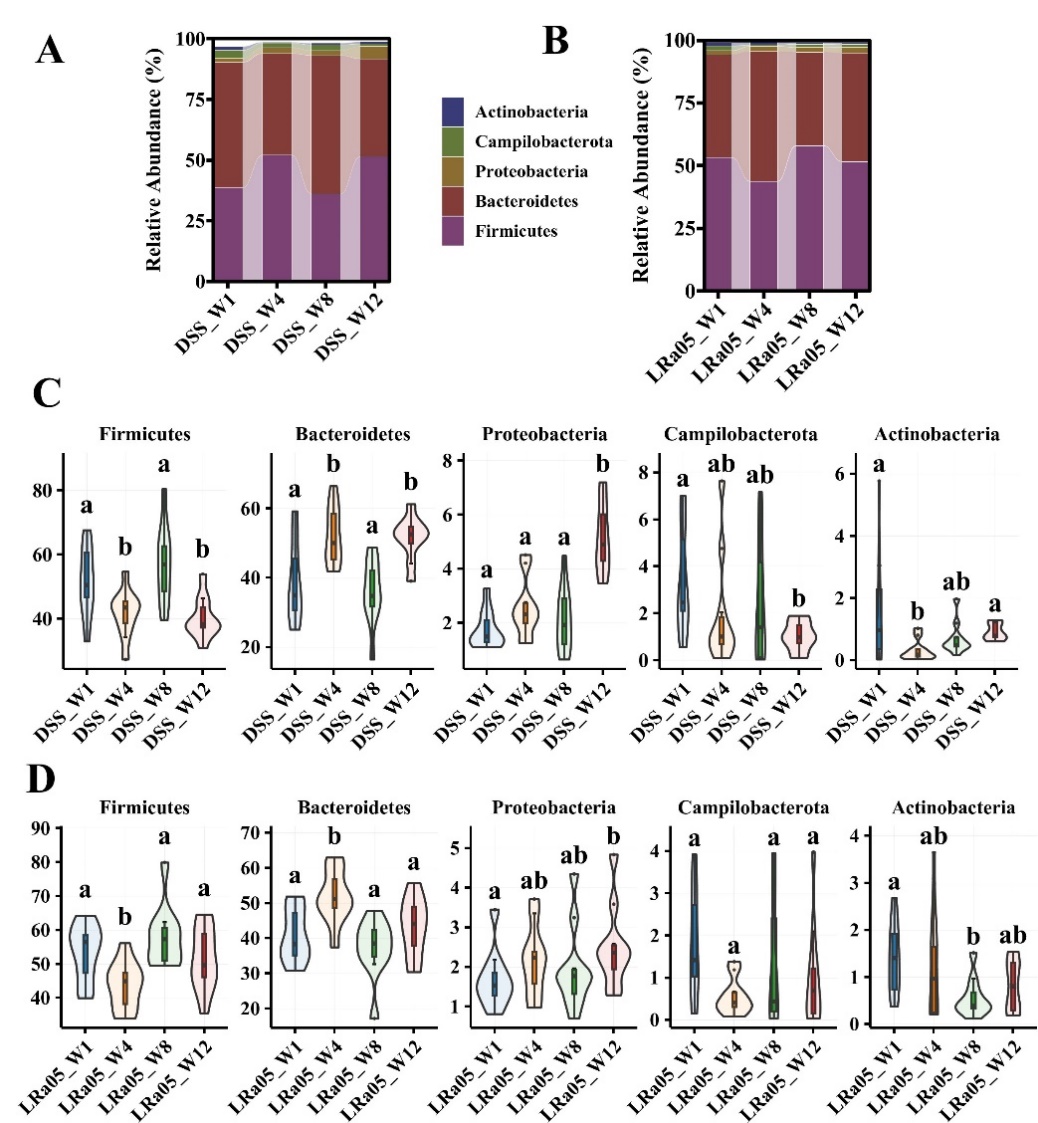


**Supplementary Figure 2.** Differential analysis of the intestinal microbiota at the phylum level between dextran sulfate sodium (DSS) and *Lacticaseibacillus* *rhamnosus* LRa05 mice at different time points. Accumulation diagram of intestinal microbiota abundance at the phylum level in DSS (A) and LRa05 (B) mice. Differences in intestinal microbiota abundance at phylum level between DSS (C) and LRa05 (D) mice at different time points. Different letters in the violin plots indicate significant differences between the group.

**Supplementary Table 1.** The tests for microbial community composition dissimilarity between pairs of groups were performed using nonparametric permutational multivariate ANOVA with the adonis2 function of vegan in R (version 4.3).

| Pairs | Df | SumsOfSqs | F.Model | R2 | p.value | p.adjusted |
| --- | --- | --- | --- | --- | --- | --- |
| CTL_W12 vs DSS_W12 | 1 | 1.256909125 | 8.116742701 | 0.310786946 | 0.001 | 0.003 |
| CTL_W12 vs LRa05_W12 | 1 | 0.914286076 | 6.364398357 | 0.261217136 | 0.001 | 0.003 |
| DSS_W12 vs LRa05_W12 | 1 | 0.809870789 | 5.428104363 | 0.231692 | 0.001 | 0.003 |

**Supplementary Table 2.** The tests for microbial community composition dissimilarity between pairs of groups were performed using nonparametric permutational multivariate ANOVA with the adonis2 function of vegan in R (version 4.3).

| Pairs | Df | SumsOfSqs | F.Model | R2 | p.value | p.adjusted |
| --- | --- | --- | --- | --- | --- | --- |
| DSS_W1 vs DSS_W4 | 1 | 0.740252525 | 4.600007012 | 0.20354007 | 0.001 | 0.006 |
| DSS_W1 vs DSS_W8 | 1 | 0.716433309 | 4.691274555 | 0.206743546 | 0.001 | 0.006 |
| DSS_W1 vs DSS_W12 | 1 | 1.378744392 | 8.377592766 | 0.317602627 | 0.001 | 0.006 |
| DSS_W4 vs DSS_W8 | 1 | 0.488560232 | 3.361533774 | 0.157363877 | 0.001 | 0.006 |
| DSS_W4 vs DSS_W12 | 1 | 1.29145407 | 8.215482111 | 0.313382835 | 0.001 | 0.006 |
| DSS_W8 vs DSS_W12 | 1 | 0.929699939 | 6.240035957 | 0.257426844 | 0.001 | 0.006 |

**Supplementary Table 3.** The tests for microbial community composition dissimilarity between pairs of groups were performed using nonparametric permutational multivariate ANOVA with the adonis2 function of vegan in R (version 4.3).

| Pairs | Df | SumsOfSqs | F.Model | R2 | p.value | p.adjusted |
| --- | --- | --- | --- | --- | --- | --- |
| LRa05_W1 vs LRa05_W4 | 1 | 0.877719786 | 8.531574501 | 0.321563068 | 0.001 | 0.006 |
| LRa05_W1 vs LRa05_W8 | 1 | 0.819537665 | 5.967733083 | 0.248990301 | 0.001 | 0.006 |
| LRa05_W1 vs LRa05_W12 | 1 | 1.12015933 | 9.241088531 | 0.339233453 | 0.001 | 0.006 |
| LRa05_W4 vs LRa05_W8 | 1 | 0.641564783 | 4.759428774 | 0.209118991 | 0.005 | 0.03 |
| LRa05_W4 vs LRa05_W12 | 1 | 0.961073065 | 8.097635469 | 0.310282343 | 0.001 | 0.006 |
| LRa05_W8 vs LRa05_W12 | 1 | 1.00056479 | 6.533883069 | 0.266320788 | 0.001 | 0.006 |
